# The Vital Role of Thanatochemistry in the Postmortem Diagnostic of Diabetic Ketoacidosis—Case Report

**DOI:** 10.3390/diagnostics11060988

**Published:** 2021-05-29

**Authors:** Nona Girlescu, Bogdan Stoica, Iuliana Hunea, Madalina Diac, Simona Irina Damian, Sofia David, Tatiana Iov, Daniel Tabian, Diana Bulgaru Iliescu

**Affiliations:** 1 Morphofunctional Sciences 1 Department, Faculty of Medicine, “Grigore T. Popa” University of Medicine and Pharmacy of Iasi, 700115 Iasi, Romania; nona-girlescu@umfiasi.ro; 2 Department of Forensic Medicine, Institute of Forensic Medicine, 700455 Iasi, Romania; zamisnicu.iuliana@gmail.com (I.H.); madalinadc89@gmail.com (M.D.); si_damian@yahoo.com (S.I.D.); ddavid.sofia@yahoo.com (S.D.); iovtatiana@yahoo.com (T.I.); bulgarudiana@yahoo.com (D.B.I.); 3 Morphofunctinal Science 2 Department, Faculty of Medicine, “Grigore T. Popa” University of Medicine and Pharmacy of Iasi, 700115 Iasi, Romania; 4 Forensic Science Department, Faculty of Medicine, “Grigore T. Popa” University of Medicine and Pharmacy of Iasi, 700115 Iasi, Romania; daniel.tabian@unitbv.ro

**Keywords:** diabetic ketoacidosis, postmortem biochemistry, glucose, ketone bodies, beta-hydroxybutyrate, acetone

## Abstract

Diabetic ketoacidosis (DKA) is a lethal acute hyperglycemic complication of diabetes mellitus (DM) and it represents the initial manifestation of DM in about 15–20% of cases in adults and about 30–40% of cases in children. Postmortem diagnosis of DKA can only be made by applying thanatochemistry. Biochemistry applied postmortem is viewed with skepticism by many practitioners in the forensic field, completely lacking in many forensic services around the world, and especially in the national ones. This article aims to underline the importance of the postmortem application of biochemistry by reviewing the case of a person in the third decade of life who died suddenly at home due to diabetic ketoacidosis (DKA), whose autopsy was performed at an early PMI of approximately 24 h. Routine postmortem examinations (macroscopic, anatomopathological, and toxicological) could not establish a clear cause of death. When attention was turned to biochemical determinations (i.e., determination of glycated hemoglobin, glucose and ketone bodies (acetone, beta-hydroxybutyrate) in the blood, vitreous humor, and cerebrospinal fluid), the identified values clarified the thanatogenic mechanisms by establishing the diagnosis of DKA.

## 1. Introduction

Diabetic ketoacidosis (DKA) is an acute hyperglycemic complication of diabetes mellitus (DM), being the leading cause of death among children and young adults with type 1 DM [1]. Although it is claimed to be specific to persons with type I DM, at least one third of cases reported with DKA have been found in people with type II DM [2]. According to the published literature, 50% of deaths in people with type I DM, under the age of 30, are due to DKA, while cardiovascular disease is among the top causes of death in those over 30 years of age [3,4,5]. The numerous precipitating causes (infections, cardio-vascular and cerebrovascular events, alcohol consumption, poor adherence to treatment, psychiatric disorders, epilepsy) that can lead to DKA must be identified immediately to prevent this lethal complication. Even so, it should be known that DKA is the initial manifestation of DM in about 15–20% of cases in adults and about 30–40% of cases in children [6,7,8].

DKA can occur when there is a relative or absolute deficiency in insulin levels, which directly relate to the elevation of counterregulatory hormone levels with insulin-antagonist properties in the liver and peripheric tissues. Thus, an exaggeration of the physiological mechanisms occurs with the triggering of a severe metabolic imbalance translated by hyperglycemia and ketosis. Postmortem morphological changes that may occur in DKA deaths are controversial since most autopsies do not identify an obvious morphological substrate to rely on. There are authors who consider Armanni–Ebstein kidney injuries as pathognomonic for DKA [9,10]. Recent studies correlate Wischnewsky gastric lesions and acute esophageal necrosis with death caused by DKA [11,12,13].

When the forensic examination is inconclusive, the diagnosis of DKA can only be established biochemically, by determining glucose and beta-hydroxybutyrate (BHB) [14]. Biochemistry applied postmortem is viewed with skepticism by many practitioners in the forensic field, and is completely absent in many forensic services around the world, especially in the national ones.

This article aims to underline the importance of the postmortem application of biochemistry by reviewing a case of a person in the third decade of life who died suddenly at home due to diabetic ketoacidosis (DKA), whose autopsy was performed at an early PMI of approximately 24 h [15]. Routine postmortem examinations in our legal medicine service (macroscopic, anatomopathological and toxicological) revealed only the presence of a small gastric hemorrhage and could not establish a clear cause of death. When attention was turned to biochemical determinations (i.e., determination of glycated hemoglobin (HbA1c), glucose, and ketone bodies (acetone, BHB) in the blood (HbA1c, acetone, BHB), vitreous humor (glucose, BHB) and cerebrospinal fluid (glucose, BHB), the identified values clarified the thanatogenic mechanisms by establishing the diagnosis of DKA.

## 2. Case Report

### 2.1. Case History

This case involves a 31-year-old man who was found deceased in his home by a neighbor. He was last seen alive the day before his death. He lived alone in an apartment in a block of flats in the city of Iaşi, Romania. The summary investigation data provided by the police revealed that the young man was living a disorderly lifestyle, being a heavy smoker, particularly of tobacco (no nature was specified). Additionally, the investigation data mentioned that he suffered from diabetes and epilepsy, diseases for which he was receiving treatment (the type of treatment was not mentioned). No medical documents (family doctor’s files, admission/discharge sheets, prescriptions of medicines) were made available to the coroner. The crime scene investigation did not reveal any aspects/elements providing further information about the circumstances of death.

### 2.2. Postmortem Findings

During autopsy, the external examination of the body revealed an asthenic constitution, of tall and slender appearance, without traumatic injuries and with the signs of death present. The PMI assessment was performed using the classical triad: rigor mortis, livor mortis, and algor mortis. Livor mortis were identified on the dorso-lateral face of the corpse, having a confluent character and did not disappear under pressure (fixed hypostasis stage). Rigor mortis was identified in all joints including the jaw and the knees, so that a stage of generalization was established. His rectal temperature on the day he was found was 22 °C, and the room temperature was 19 °C. No signs of putrefaction were identified. The PMI was set to be approximately 24 h. The internal examination of the body, carried out by thorough macroscopic examination of each individual viscera, both on the surface and in cross-sections, identified only a generalized visceral stasis and the presence in the gastric lumen of 200 mL of dark red fluid blood, without alterations of the gastric mucosa. Otherwise, all organs were without visible macroscopic changes. Organ fragments, blood, urine, vitreous humor (VH), and cerebrospinal fluid (CSF) were collected for complementary examinations. The organ fragments were initially fixed in formaldehyde and subsequently processed. Blood was collected from the periphery (femoral vein) and from a central source (left heart). Urine was collected by puncturing the anterior wall of the bladder. VH was collected from both eyes with a 10 mL syringe and a 20-gauge needle by puncturing the lateral cantus of the eyeball. The CSF was collected from two places: suboccipital and lumbar. Through suboccipital puncture, CSF was collected from the cerebellum–medullary cistern. Using a lumbar puncture, CSF was harvested from the spinal subarachnoid space corresponding to the L4–L5 vertebrae.

The histopathological examination carried out on the collected tissue fragments from each organ did not identify any notable morphological changes that could help identify a possible cause of death, only confirming the generalized visceral stasis also found during the macroscopic examination. No specific lesions were identified. At both macro and microscopic examination of the pancreas, only interstitial congestion and autolytic changes were identified: venous type vessels at the level of interlobular septa with dilated lumens and full of red blood cells, pancreatic acinar cells with focal disappearance of nuclei. The general toxicological examination, which aimed to identify ethanol, methanol, isopropanol, drugs, pesticides, organophosphates, and ethylene glycol was negative. On the other hand, the gas-chromatographic method (Agilent 6080 N—Agilent Technologies Inc., Wilgminton, NC, USA), which sought to identify alcohols, revealed the presence of acetone (Ac) in a concentration of 89.53 mmol/L (0.52 g/L).

### 2.3. Biochemical Analyses

The biochemical analyses focused on identifying glucose, ketone bodies (Ac, BHB), and HbA1c. Glucose was determined from three sites: VH, cerebellar-medullary CSF, and lumbar CSF. Ac was determined from peripheral blood. BHB was determined from peripheral blood, VH, cerebellar-medullary CSF, and lumbar CSF. HbA1c was determined from peripheral blood and central blood.

### 2.4. Glucose Method

Glucose determination was performed using a glucose GOD-PAP Kit according to the manufacturer’s protocol (Biosystems, Barcelona, Spain). This end-point method is based on hydrogen peroxide generation from the interaction of glucose–glucose oxidase, coupled with the generation of a red quinoneimine dye. The absorbance was determined at 546 nm in a biochemistry analyzer (Piccos-AMP Diagnostics, Graz, Austria).

### 2.5. BHB Methods

The determination of BHB was carried out by two methods.

The first used a commercially available Multi-Functional Monitoring System XPER Technology analyzer (TaiDoc Tech Corporation, New Taipei City, Taiwan), with its specific test strips for BHB, acquired as part of doctoral research. Before the determination, a control solution test was performed according to the manufacturer’s instructions. The blood BHB was based on the measurement of electrical current generated by the reaction of blood BHB with the reagent on the strip. The measuring range for BHB is 0–8.0 mmol/L.

The second method was based on the use of a Beta-Hydroxybutyrate Assay Kit (MAK041, Sigma-Aldrich, Saint Louis, MO, USA) acquired during doctoral research. In this kit, BHB concentration is determined by the generation of a colorimetric (450 nm) product, proportional to the β-Hydroxybutyrate present. The end-point reading was performed using a Platos microplate reader (AMP Diagnostics, Graz, Austria).

### 2.6. Ac Method

The determination of Ac was carried out within the Iasi Toxicology Laboratory of the Institute of Legal Medicine by the gas-chromatographic method (Agilent 6080 N, head space unit Agilent 7697) routinely used for the determination of alcohols, with a detection limit for Ac of 17.21 mmol/L (0.1 g/L).

### 2.7. HbA1c Method

The determination of HbA1c was made with an automatic analyzer (HemoCue HbA1c 501—Radiometer, Danemarca) acquired as part of doctoral research, with the method used based on the fully automated boronate affinity assay, with the percentage determination of HbA1c ranging between 4.0–14.0% (NGSP). The analyzer was certified by the National Glycohemoglobin Standardization Program (NGSP)/Diabetes Control and Complications Trial (DCCT) and calibrated according to the standards developed by the International Federation of Clinical Chemistry (IFCC).

The results are presented in Table 1.

### 2.8. Postmortem Diagnostic Criteria for DM

According to the International Expert Committee, the American Diabetes Association (ADA) and the World Health Organization (WHO), the diagnosis of DM can be established when HbA1c values are ≥6.5% (≥48 mmol/mol) [25,26,27].

### 2.9. Postmortem Diagnostic Criteria for DKA

Postmortem diagnostic criteria for DKA include increased glucose values in VH and/or CSF >10 mmol/L (>180 mg/dL) [16,17,18,19,20] and ketone bodies value increase five times above the normal threshold (normal values differ depending on the method of determination); for the colorimetric method, using the MAK041 kit, the diagnostic threshold value is 0.5 mmol/L [28]; for the point-of-care system XPER Technology, the diagnostic threshold value is 2.5 mmol/L [14,16,17,18,19,20,21,22,23,24].

### 2.10. Cause of Death

Corroborating all these data from macroscopic and microscopic examinations, the negative toxicological examination, the postmortem confirmation of an uncontrolled DM, postmortem identification of the state of ketoacidosis expressed by hyperglycemia (increased glucose levels harvested from three sites above >10 mmol/L), and ketoacidosis (increased levels of Ac and BHB) led to the diagnosis of death by DKA.

## 3. Discussion

Death due to DKA can have important forensic implications, so it is imperative that the postmortem diagnosis can be substantiated. The classical autopsy performed on a person who died of DKA cannot provide sufficient data on the thanatogenic mechanism because this pathology does not present an obvious morphological substrate. It does not matter how complete the macroscopic, histopathological, and toxicological examinations are as they fail to identify the cause of death. In this respect, the attention of the coroner must necessarily turn to thanatochemical investigations [14,27,29,30]. The collection of bodily fluids, capable of providing reliable information on the values of DKA specific markers, is mandatory. It is obvious that if this autopsy step is skipped, the diagnosis of DKA can no longer be substantiated.

Thanatochemical determinations must take into account the changes that occur postmortem (i.e., stopping active membrane transport, loss of selective membrane permeability, cessation of metabolic activity), ideally being performed in an early post-mortem interval (PMI) before putrefaction [14,15]. An early PMI is defined as a death occurring fewer than 72 h before the postmortem [15]. Regarding the postmortem imbalances of carbohydrate metabolism, it should be noted that postmortem determination of blood glucose is unreliable, with significant fluctuations due to postmortem hemolysis and anaerobic glycolysis [14,16]. The highest concentrations are found in blood collected from the left heart, vena cava or hepatic vein, most likely due to hepatic glycogenolysis [14,16]. Because of this, other fluids have been proposed for glucose determination such as vitreous humor (VH) and cerebrospinal fluid (CSF) [14,16,17,18,19,20]. VH is considered the fluid of choice, being the most protected site against postmortem changes, with the lowest rate of glycolysis. The second recommended site for glucose determination would be CSF, being well correlated with VH values [14,16,17,18,19,20]. The vitreous glucose concentration is considered to be elevated when it exceeds 6.9–10 mmol/L (the threshold depending on the study), corresponding to an antemortem hyperglycemia prior to death [14,16,17,18,19,20]. BHB is the main compound responsible for the elevated anion gap in ketotic cases, representing approximately 78% of the total ketone body [18]. The latest published literature data show that it is a fairly stable postmortem marker, and can be determined from both blood and other body fluids like VH and CSF [17,18,19,20,21,22,23]. According to our case studies and literature data, the postmortem diagnosis of DKA is established by the concomitant presence of hyperglycemia (elevated glucose levels greater than 10 mmol/L in VH and/or CSF) and ketoacidosis (BHB levels greater than 2.5 mmol/L in blood/VH/pericardial fluid/CSF) [14,16,17,18,19,20,21,22,23]. Another useful marker in demonstrating carbohydrate imbalance is glycated hemoglobin (HbA1c), which provides information on glycemic status 8–12 weeks prior to testing. HbA1c can be determined only from the blood, corresponds well to antemortem values, and shows good stability in the early phase of PMI [14,18,25].

In the case described, by corroborating the available data, although minimal, a causal hypothesis could be outlined, with the DKA in the forefront. The following criteria had to be met to confirm the hypothesis: confirmation of DM and/or the presence of uncontrolled glycemic status, proof of the presence of hyperglycemia and ketoacidosis immediately prior to death. The absence of identifiable traumatic or pathological changes during autopsy combined with the mere mention of the diagnosis of DM in the investigation data (with the absence of any medical documents) in a young person led to the decision to harvest the bodily fluids needed to identify an imbalance of the carbohydrate metabolism with lethal potential.

The initial indication that these chemical investigations were appropriate was the postmortem confirmation of DM merely mentioned in the investigation data by determining the HbA1c in the deceased’s blood. It is stated that HbA1c represents a helpful post-mortem tool in identifying undiagnosed diabetes as well as in differentiating DKA from alcoholic ketoacidosis [28,29,30]. This marker corresponds well to antemortem values, and it seems that it is stable after death for at least 36 h [16,25]. In order to determine an accurate value, any interference that could affect its value (i.e., anemia, splenectomy, polycythemia, pregnancy) must be excluded [16,24]. In this case, they were excluded. Thus, at a PMI of approximately 24 h, we identified an HbA1c value of 12.6% in peripheral femoral blood, respectively 12% in left heart (central) blood, which confirmed the diagnosis of DM. Moreover, an uncontrolled and unbalanced DM was identified by a HbA1c value almost double the target threshold [14,28].

Another essential aspect of the case is that the VH is considered the fluid of choice for postmortem analysis of glucose, being more protected from the degradation processes that occur following death [14,18,19]. VH glucose corresponds to 50–85% of blood glucose [17,18,19]. In the first six hours postmortem, glycolysis causes a decrease in VH glucose concentration by 35–70%, then remains stable for several days [17]. For this reason, real hypoglycemia can hardly be diagnosed postmortem. Regarding hyperglycemia, the postmortem diagnosis can be sustained if a diagnostic threshold value is identified, respectively higher than 10 mmol/L, which theoretically corresponds to an antemortem blood glucose concentration of approximately 26 mmol/L (468 mg/dL) [14,16,17,18,19,20]. Some authors have proposed the inclusion of lactate in the diagnosis of hyperglycemia (Traub’s formula), but these studies are controversial and offer little credibility because lactate can be produced postmortem [16,17,18,19,20]. CSF could be an alternative to VH [14], especially when the latter cannot be harvested (such as the absence of eyeballs or ophthalmological pathologies whereas the vitreous body is also affected). In our case, both VH and CSF (from two sites each) were harvested. The glucose values identified in the VH (230 mg/dl) and CSF (CSF O 218 mg/dl and CSF L 202 mg/dl) confirmed hyperglycemia immediately before death. These three glucose levels are consistent with the literature, the phenomenon of glycolysis being more pronounced in the CSF, thus with lower detectable values in this fluid [14,19,20]. It should be noted that elevated postmortem glucose levels may exist in other situations such as asphyxia, electrocution, hypothermia, and congestive heart failure [29,30,31,32,33]. These causes must be excluded in order to support a hypothesis of hyperglycemia before death, as was done in the present case.

The postmortem diagnosis of ketoacidosis is established by detecting and identifying elevated levels of ketone bodies [14,16,20,21,22,23]. Ali Z et al. stated in 2012 that the mere identification of Ac levels above 0.1 g/L can diagnose DKA when hyperglycemia is present [30]. Other authors have stated that Ac can only be considered as a screening marker, helping decide whether to determine the BHB level or not [23]. A study recently published in 2021 states that postmortem determination of BHB should be carried out routinely in all forensic cases [23]. BHB has proven to be the most faithful ketone body for ketoacidosis, and can increase in DKA, but also in alcoholic ketoacidosis or hypothermia [23,25,34,35]. Recent studies show that BHB is stable postmortem, unlike Ac or isopropanol, which can increase postmortem in advanced cases of decomposition; the latter two may also have an exogenous origin [34,35]. In this case, the search for ketone bodies was carried out in three bodily fluids: blood, VH, and CSF. The Ac was identified in the blood of the deceased at a value of 0.5 g/L, five times the detection limit (0.1 g/L). The determination of blood BHB level was carried out with a point-of-care monitoring device, with the resulting values above 2.5 mmol/L establishing the diagnosis of ketoacidosis [24]. The BHB value in the blood of the deceased was 7.3 mmol/L, thus almost three times the diagnostic threshold value. The BHB level was also determined quantitatively in VH and CSF, using a research kit, the diagnostic threshold value of which was considered 0.5 mmol/L [28]. The threshold value of 0.5 mmol/L was established by referencing the values considered normal, obtained in the control cases studied with this kit (less than 0.1 mmol/L, thus at least five times lower) and also by referencing other studies using the same kit [28]. In the case of these determinations, the BHB values were significantly increased (see Table 1), clearly supporting the state of ketoacidosis in the person at the time of death.

In the present case, initially considered as a case of unexplained death, the three concurring postmortem markers (HbA1c, glucose, BHB) reconfirmed the antemortem clinical diagnosis of DM and furthermore established DKA as being the main cause of death.

It should be noted that the lowest value for BHB, colorimetrically identified using the MAK041 kit, was found in the CSF harvested by lumbar puncture, the value being almost two times lower than the other two BHB values in VH and CSF O (see Table 1). We have not been able to find a concrete explanation for the differences in BHB values in the three sites (VH, CSF O, CSF L). As the glucose level identified in the CSF L was also the lowest, we do not recommend that CSF L be considered as a preferred site for postmortem determinations of glucose and BHB.

Although considered pathognomonic for DKA by some authors [9,10], Armanni–Ebstein renal lesions were not identified in this case. The blood identified in the gastric lumen, the only obvious morphological change, may have two explanations. Either a synergistic effect of the DKA induced acidosis, with the manifestation of coagulation disorders and subsequent hemorrhage, or the existence of a marked physiological stress often present in agonic states [12]. Wischnewsky gastric lesions and acute esophageal necrosis were also not identified in our case. The presence of hemorrhage in the gastric lumen is at least interesting, and we have the courage to say that if death would have occurred later, there would have been the possibility for the Wischnewsky spots to appear or even for acute esophageal necrosis to develop [12,13]. Many autopsies performed on DKA cases that have been reported in the literature speak about the absence of characteristic macro and microscopic changes [6,36]. Additionally, in this case, the pancreas was characterized only by autolysis. It is known that the specific DM lesions (reduction in the number and size of pancreatic islets or aspects of autoimmune islet-inflammatory mononuclear infiltrate in the Langerhans Islands) may be unsteady and absent when the condition becomes clinically manifest, as most likely happened in the present case [37]. Moreover, it should be emphasized that the described changes focally affect the pancreatic endocrine component [37]. This is where the importance of continuing autopsy steps with thanatochemical investigations lies.

Aside from the classical quantitative biochemical determinations made postmortem in the different fluids of human body, there are also studies on animal models that have applied a metabolomic approach, for example, by analyzing the metabolic profile of VH and aqueous humor at different times after death [38,39]. It seems that the H-NMR analysis used provides a more detailed picture of the changes that occur postmortem [40]. The results obtained in the present case could be interpreted in a metabolic context in which a large number of analytes are taken into account, able to provide much more accurate information including those pathological changes that otherwise can be easily overlooked.

## 4. Conclusions

The postmortem diagnosis of DKA can be a real challenge for the coroner and knowing and following the complementary autopsy steps is mandatory. Moreover, it should be pointed out that thanatochemistry is not as accessible to legal medicine services as other autopsy examinations (macro and microscopic, toxicological investigations), being known that it is not routinely carried out or even not at all in some legal medicine services.

The reporting of this case aims to emphasize the imperative role that thanatochemistry plays in legal medicine, especially in cases of deaths due to DKA. The collection of suitable biological fluids, the correct determination of specific biomarkers, and the correlation and interpretation of the resulting values, ultimately supported the diagnosis of DKA.

## Figures and Tables

**Table 1:** Results for the biochemical parameters studied.

Parameter	Obtained Value	Reference Value
Glucose in vitreous humor	12.765 mmol/L (230 mg/dL)	hyperglycemic state >10 mmol/L (>180 mg/dL) [16,17,18,19,20]
Glucose in occipital cerebrospinal fluid (CSF O)	12.099 mmol/L (218 mg/dL)	hyperglycemic state >10 mmol/L (>180 mg/dL) [16,17,18,19,20]
Glucose in lumbar cerebrospinal fluid (CSF L)	11.211 mmol/L (202 mg/dL)	hyperglycemic state >10 mmol/L (>180 mg/dL) [16,17,18,19,20]
Acetone (Ac) in blood	0.52 g‰	negative [14]
Beta-hydroxybutyrate (BHB) in vitreous humor (VH)	0.57 mmol/L	negative [14]
Beta-hydroxybutyrate (BHB) in occipital cerebrospinal fluid (CSF O)	0.55 mmol/L	negative [14]
Beta-hydroxybutyrate (BHB) in lumbar cerebrospinal fluid (CSF L)	0.2986 mmol/L	negative [14]
Beta-hydroxybutyrate (BHB) in blood (XPER Technology analyzer)	7.3 mmol/L	negative [14] >1.5 mmol/L risk of developing DKA Ketoacidosis >2.5 mmol/L [14,16,17,18,19,20,21,22,23,24]
Glycated hemoglobin (HbA1c) in peripheral blood	12.6%	normal 4.8–5.6%; prediabetes 5.7–6.4%; diabetes ≥ 6.5%; the therapeutic target for diabetics is 7% [25,26,27]
Glycated hemoglobin (HbA1c) in central blood	12%	normal 4.8–5.6%; prediabetes 5.7–6.4%; diabetes ≥6.5%; the therapeutic target for diabetics is 7% [25,26,27]

## Data Availability

This article did not report any data, apart from those reported here.
